# Effectiveness of Osteopathic Treatment in Adults with Short Hamstring Syndrome: A Systematic Review

**DOI:** 10.3390/jcm13206076

**Published:** 2024-10-12

**Authors:** Hugo Ogando-Berea, Raquel Leirós-Rodríguez, Pablo Hernandez-Lucas, Óscar Rodríguez-González

**Affiliations:** 1 LabEndo Research Group, Department of Functional Biology and Health Sciences, University of Vigo, Campus Lagoas-Marcosende, 36310 Vigo, Spain; hoberea@uvigo.gal; 2 SALBIS Research Group, Nursing and Physical Therapy Department, University of León, Astorga Ave., 24401 Ponferrada, Spain; rleir@unileon.es; 3 Department of Functional Biology and Health Sciences, Faculty of Physiotherapy, University of Vigo, Campus A Xunqueira, 36005 Pontevedra, Spain; panderetu@hotmail.com

**Keywords:** hamstring muscles, musculoskeletal manipulations, osteopathic manipulation, osteopathic medicine rehabilitation, osteopathy, manual therapy interventions, physiotherapy

## Abstract

**Background/Objective**: Short hamstring syndrome is common in the general population and can lead to impaired balance, function, and posture, and increased risk of injuries. Local treatments have obtained controversial results, so it is necessary to evaluate the effectiveness of other types of therapy such as osteopathic treatment. To evaluate the efficacy of osteopathic techniques in increasing the elasticity of the hamstring musculature in short hamstring syndrome. **Methods**: A systematic review of randomised controlled trials was conducted in PubMed, Medline, Cinhal, Scopus, WOS, SPORTDiscuss, and PEDro. The PEDro scale was used to evaluate the methodological quality and the RoB2 for the evaluation of biases. **Results**: A total of eight articles were selected. Most of the participants were assessed with the Active Knee Extension or Straight Leg Raise tests. The osteopathic techniques used were the muscle energy technique, suboccipital inhibition, and vertebral mobilisations. As for the control interventions, they mainly included passive stretching and placebo. **Conclusions**: The results suggest that osteopathic techniques are more effective than placebo or other interventions in increasing flexibility in adult patients with short hamstring syndrome. This effect can be explained by neurophysiological (Golgi apparatus, neuromuscular spindle activity, and Hoffmann reflex) and structural factors (dura mater, posture, and myofascial chains). Nevertheless, the evidence suggests that it would be beneficial to incorporate this type of treatment into flexibility improvement programmes.

## 1. Introduction

Short hamstring syndrome (SHS) is a condition characterised by a reduction in extensibility or shortening of the hamstring muscles [1,2]. Although the exact causes of SHS are not clearly defined in the scientific literature, some researchers link it to the hamstring’s multi-articular function, tonic postural nature, and continuous exposure to significant tensional forces [3]. Other associated risk factors include male gender [1,4], postural alterations [5], sports practice [6,7], and situations such as the work environment or prolonged sitting [8]. The most commonly used tests to measure hamstring flexibility are the active knee extension (AKE) test, straight leg raise (SLR), and popliteal angle (AP) test, all of which provide valuable information on hamstring flexibility and have good inter-rater reliability, due to the influence of a single joint during their performance [9,10,11,12]. Ferrer [4] established a classification of SHS into two levels for the SLR test (type I shortening between 61° and 74° and type II shortening at gradations below 60°) and for the AP test (type I shortening between 16° and 34° and type II shortening at gradations greater than 35°) [4].

Several authors had established an association between the decreased extensibility of the hamstring musculature with other health problems such as those predisposing athletes to muscle injuries [13], non-specific back pain [14,15], low back pain [1,2,15,16,17,18], patellar tendinopathy [19,20], biceps femoris ruptures [21], and patellofemoral syndrome [22,23].

Given the potential impact of hamstring shortening on distal areas such as the spine, it is important to adopt a holistic perspective in the management of SHS [15,18,23,24]. This approach is based on the theory of tensegrity, which provides a theoretical framework for understanding the functional interconnection between different fascial segments of the body [25]. Applied to SHS, this theory explains how musculoskeletal structures, including fascia and muscles, work together to maintain bodily balance and stability. Tensegrity suggests that changes in one area of the body can affect other areas due to the interrelationship of the myofascial structures. In the case of hamstring shortening, this could generate tension along the posterior chain, affecting the lumbar region, sacrum, and even cervical structures, thereby altering overall postural alignment [25,26]. Therefore, the theory of tensegrity suggests that SHS treatment should not be limited solely to the hamstrings. In this line, several investigations have obtained beneficial effects in the treatment of SHS with non-direct techniques on the hamstring musculature, for example through osteopathic treatments focused on the inhibition of remote regions such as the masseters [25] or the diaphragm [27].

There are previous reviews that have investigated the effectiveness of stretching in increasing flexibility in healthy young adults [28,29]. While these studies have provided valuable insights into traditional stretching methods, no systematic review has been conducted on the effects of osteopathic treatment on short hamstring syndrome (SHS). Unlike conventional stretching, this approach adopts a holistic perspective, aiming to improve hamstring flexibility by considering the interconnections between different body structures. Despite its common use, the effectiveness of this approach in treating SHS has not yet been reviewed in the academic literature [25,26,30]. Therefore, the aim of this systematic review was to investigate the impact of osteopathic techniques on hamstring flexibility in adults with SHS. The hypothesis on which this review was based was that osteopathic techniques positively affect SHS, thereby increasing flexibility.

## 2. Materials and Methods

### 2.1. Data Sources and Searches

This study was registered prospectively on PROSPERO (code: CRD42023399254) and conformed to the Preferred Reporting Items for Systematic Reviews and Meta-analyses (PRISMA) [31] and the guidelines for implementing PRISMA in Exercise, Rehabilitation, Sport medicine and SporTs science (PERSIST). The PRISMA checklist is attached as Supplementary Materials, Table S1. The PICOS question was then chosen as follows: P—population: adults with SHS; I—intervention: osteopathic techniques; C—control: another intervention; O—outcome: effects on hamstring flexibility; S—study designs: randomised controlled trial.

A systematic search for randomised controlled trials was conducted in December 2023 across seven databases: PubMed, Medline, Cinahl, Scopus, Web of Science, SPORTDiscus, and PEDro. The search strategy, aimed at addressing the PICOS question, is detailed in the Supplementary Materials, Table S2. For this purpose, the following Medical Subject Headings (MeSH) were combined: Hamstring tendons, Hamstring muscles, Chiropractic manipulation, Orthopedic manipulation, Osteopathic manipulation, Spinal manipulation, Manual therapy, Musculoskeletal manipulation, Osteopathic medicine, and Osteopath. In the PEDro database, an advanced search was performed using the keyword Hamstring and selecting the option Stretching, Mobilisation, Manipulation.

### 2.2. Study Selection

After removing duplicates, two reviewers independently assessed the articles for eligibility. In cases of disagreement, a third reviewer made the final decision on whether the study should be included. The following inclusion criteria were applied for study selection: (i) randomised clinical trials; (ii) studies that assessed the efficacy of any osteopathic treatment technique on hamstring group elasticity; (iii) studies having participants with a limitation of at least 15° in the popliteal angle in a test measuring the extensibility of the hamstring muscles; (iv) studies in English or Spanish. Conversely, the following studies were excluded from this review: (i) studies evaluating subjects with hamstring pathology other than the above or with other associated pathology; (ii) studies with participants under 18 years of age or over 65 years of age. After screening the data and extracting titles and abstracts based on the inclusion criteria, the selected abstracts were retrieved in full text. Titles and abstracts that lacked sufficient information regarding the inclusion criteria were also obtained in full text. Two reviewers then selected full-text articles that met the inclusion criteria, using a data extraction form. Both reviewers independently extracted data from the included studies using a customised data extraction table in Microsoft Excel 365. In cases of disagreement, the reviewers discussed the matter until a consensus was reached.

### 2.3. Data Extraction and Quality Assessment

The following data were extracted for further analysis: demographic details (title, authors, journal, and year), sample characteristics (age, sex, inclusion and exclusion criteria, and number of participants), and study-specific parameters (duration of the intervention, adverse events, and type of treatment technique). Tables were used to summarise both the study characteristics and the extracted data. The quality of the studies was assessed according to the PEDro scale, while the Rob2 tool was used to evaluate the risk of bias in the studies included in this review.

## 3. Results

### 3.1. Included Studies

**Figure 1:**
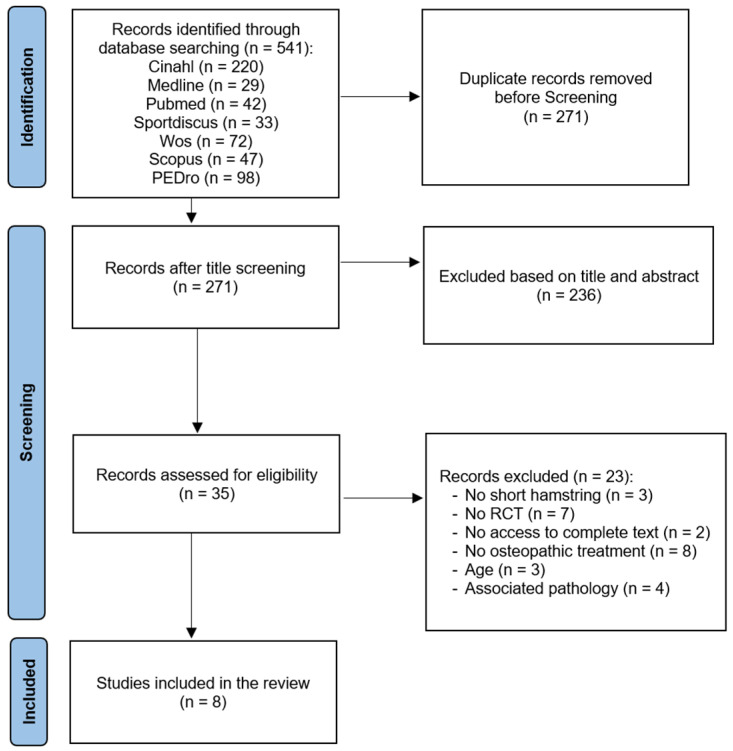
PRISMA flow diagram.

The first search returned 541 results and, after eliminating duplicates, 271 studies were considered valid for inclusion. Of the 271 papers reviewed, 236 were excluded after review of the abstract and title. After the first reading of all full-text candidates, the Kappa score from reviewers 1 and 2 was 0.9, indicating that the set was almost perfect [32]. Finally, eight full-text articles were included in the study (Figure 1).

### 3.2. Methodological Quality of the Studies

The methodological quality of the studies was 4.8 on average (Table 1). According to the PEDro scale, the studies had a fair methodological quality [33]. Although all studies scored four or more points on this scale, the most frequent methodological flaw was the absence of blinding [34,35,36,37,38,39,40,41].

### 3.3. Risk of Bias

The risk of bias analysis using the Rob2 tool showed considerable variability across the included studies. Most studies demonstrated a low risk of bias in the ‘random sequence generation’ and ‘deviations from intended interventions’ categories. However, some concerns were identified in the ‘measurement of the outcome’, with three studies [35,38,39] being rated with a moderate risk. Additionally, in the ‘selection of the reported result’ category, all studies showed a low risk. Overall, three studies were considered to have a low risk of bias [34,36,37], while Azizi et al. [38] was rated as having a high risk due to concerns regarding the outcome measurement. These results suggest that while most studies are methodologically sound, some exhibit limitations in the measurement and handling of outcomes, which may impact the validity of the findings. Detailed bias analysis is presented in Table 2.

### 3.4. Participants

A total of 532 participants participated in the eight studies, with a mean age of 33 years. Of the participants, 51.7% were women. In three of the articles, the sex of the participants was not specified [34,38,40] (Table 3). None of the participants experienced adverse effects [34,35,36,37,38,39,40,41].

#### Procedures

The most used osteopathic interventions were muscle energy techniques [38,41] and vertebral mobilisations [34,35,36,40]. Other authors applied myofascial treatment [37,39] and active release techniques [39,41] (Table 3).

The investigations that applied muscle energy techniques employed post-isometric relaxation or Lewit method [38,41]. The post-isometric relaxation was performed by extending the subject’s knee to the point where they first experienced discomfort in the hamstring. At this point, a moderate isometric contraction (approximately 75% of maximal effort) of the hamstring muscle was induced for a duration of five seconds. After a three-second relaxation period, the technique was repeated three times, resulting in a total of four contractions [41]. Then, the patient was asked to lightly push their leg against the researcher’s unyielding counter-force, for 5 s, then relax for 10 s while the stretch was maintained [41]. The Lewit method was performed to increase hamstring flexibility [38]. Each participant was positioned in a supine position, with the hip fixed at 90 degrees of flexion, and the knee joint was passively extended until reaching the restrictive barrier. The participant was then instructed to apply pressure in the direction of knee flexion, using 75% of their maximal voluntary contraction against the therapist’s applied force, holding the contraction for 7–10 s. After relaxing the muscle, the therapist passively moved the knee into a new range of extension. This technique was repeated three times, with intervals of approximately 10 s between each repetition [38].

Interventions based on vertebral mobilisation focused on the lumbar region [35,36,37]. Two types of articulatory techniques on the lumbar region were studied in five different experimental groups [34,35,36,40]. In one study, there were two experimental groups [35]. The researchers applied central posterior–anterior mobilisation of L5 to one group [35]. To the other group, they applied lumbar mobilisations to the unilateral posterior–anterior zygapophyseal L4/L5 joint ipsilateral to the dominant limb, determined by preferred kicking foot [35]. In both experimental groups, the therapists applied grade-three mobilisations three times for two minutes at a frequency of 1 Hz [35]. This last procedure was applied to the experimental group of another study by Chesterton et al. [40], also three times for two minutes but at a 2 Hz frequency (maintained by a metronome to provide sympathetic nervous system excitability). Chesterton and Payton [34] also applied central L4 and L5 mobilisation. For this, participants lay prone on a plinth that was positioned on two force plates designed to measure the mobilisation force. During the grade-three mobilisation protocol, an average force of 104.18 ± 11.2 N was applied. The procedure was carried out for two minutes, repeated three times, alternating between L4 and L5, without any rest in between, and at a frequency of 1 Hz, which was maintained using a metronome [34]. Furthermore, force plate data were recorded at 500 Hz above the frequency of the mobilisations, preventing sampling errors [34]. Finally, Szlezak et al. [36] also employed unilaterally applied grade-three oscillatory posterior–anterior zygapophyseal mobilisations at a frequency of 2 Hz to the T12/L1, L1/L2, L2/L3, L3/L4, L4/L5, and L5/S1 for 30 s per joint (3 min total treatment), ipsilateral to the tested leg [36]. This latter investigation, in addition to the intervention group described above, had another group in which participants received static stretch of the muscles of the posterior chain (ipsilateral to the tested leg) for 3 min at the point of R1 [36].

The investigations that based their intervention on myofascial therapies were suboccipital inhibition [37,39] and bilateral myofascial release in plantar fascia [39]. Joshi et al. [39] included two experimental groups: both received both techniques (one by a therapist and another self-applied).

The suboccipital inhibition technique was applied for 2 min with the patient in the supine position and the eyes closed [37,39]. The therapist sat behind the subject’s head and placed the palms of their hands underneath it, with their fingertips resting on the posterior arch of the atlas. They applied upward pressure towards themselves, maintaining it for two minutes until tissue relaxation was achieved [37,39]. Joshi et al. [39] also applied this technique to a second experimental group with self-applied treatment. For this, participants were provided with a tool resembling a peanut-shaped lacrosse ball, created by taping two tennis balls together. They were instructed to stand against a wall and place the tool in the suboccipital region. To perform the procedure, participants were asked to tuck their chin in and, while maintaining pressure, move the tool in an upward and downward direction to cover the entire suboccipital area. This was done for two minutes and repeated once daily for two weeks [39].

The bilateral myofascial release in plantar fascia was applied in two different ways: by a therapist and self-applied [39]. In the first case, the participants lay in a prone position with feet off the edge of the couch [39]. The therapist stood at the foot of the couch, using their knuckles to engage the soft tissues at the calcaneal attachment of the plantar fascia, applying firm pressure downward towards the ball of the foot, maintaining the pressure throughout the duration of the technique. This release sequence was performed for two minutes and then repeated on the opposite foot [39]. Participants in the self-applied group were given a tennis ball and instructed to sit on a chair with the ball placed under their foot. Leaning forward, they were asked to apply pressure on the ball and roll it back and forth along the entire medial arch of the foot for two minutes, with emphasis on maintaining consistent pressure. The same procedure was repeated for the other foot and was performed once daily [39].

The most used interventions in the control group were placebo [35,36,37,38], passive stretching [39], the active release technique [41], and, in one of the articles, whole body vibration [38] or no intervention [40].

Aparicio et al. [37] applied a placebo joint articulation technique to the nose bones for 2 min. With the patients lying in a supine position and the therapist seated facing them, the therapist placed one hand on the frontal bone and the other hand on the nasal bones. The therapist then applied a downward pull while gently moving the nasal bones laterally [37]. In other studies, the placebo intervention consisted in lying prone on a plinth for a 10 [35]- or 20 [34]-minute period.

The whole-body vibration was applied with the Powerplate vibration platform for 30 s three times at 30 Hz [38]. The participants stood on the platform without shoes and socks, with bent knees (about 20 degrees) and their legs open to shoulder width, and underwent the vibration at a frequency of 30 Hz and 2 mm of amplitude. This positioning prevents vibration transfer to the head. Each participant was treated in three 30 s sets with 30 s of rest between each set [38].

The active release technique on the hamstring of the dominant side was applied in one study [41]. Subjects received a single session of this technique, which includes three steps. The subjects lay supine on the plinth, and gentle tension was applied along the entire length of the hamstring muscle while the leg was stretched in various positions to more effectively target the muscle. The tension was applied specifically at the origin and insertion points of the hamstring. Finally, gentle pressure was also applied around the adductor and gluteal muscles, as the hamstring is connected to these muscles, which could contribute to the tightness in the hamstring [41].

Finally, Joshi et al. [39] applied a bilateral static passive hamstring stretch (30 s), in which the participant lies in a supine position with head in neutral and hands by the sides. Straps were used to stabilise the contralateral leg and pelvis to the plinth. To carry out the static stretch, the hip and knee were positioned at 90 degrees, and the knee was gradually extended until the therapist encountered maximum resistance. The stretch was held for 30 s and repeated three times, with a 15 s rest interval between each repetition [39].

### 3.5. Results for Hamstring Flexibility

The muscle energy techniques [38,41], suboccipital inhibition [37,39], joint mobilisations [34,35,36,40], and the active release technique [41,42] obtained significant improvements after their application. The approaches based on muscle energy techniques (post-isometric relaxation [41,43] and Lewit method [38]) showed significantly positive results from baseline to post-intervention.

Post-isometric relaxation performed significantly worse than the active release technique with which it was compared [41]. In the case of the Lewit method, there were no significant differences in its comparison with the whole-body vibration treatment with which it was compared [38].

Suboccipital inhibition showed significant effects in all the applications performed, both alone [37] and when applied together with myofascial relaxation of the plantar fascia [39]. It also showed positive effects when compared with placebo [37]. Additional benefits were observed when this technique was combined with plantar fascia myofascial relaxation and passive stretching, compared to passive stretching alone and with suboccipital inhibition plus plantar fascia myofascial relaxation [39].

Regarding the techniques based on active release, the one based on elongation with fascial sliding traces was statistically superior to the muscle energy techniques with which it was compared [41].

In the control groups, none of the placebos used presented significant post-intervention improvement [34,35,36,37,40], and in the case of passive stretching, significant improvements were found when they were performed for 30 s [39] but not when they were performed for 3 min [36].

In summary, the results showed that several osteopathic techniques achieved significant improvements. Suboccipital inhibition and muscle energy techniques were effective, though post-isometric relaxation was less effective than active release. The Lewit method showed no differences compared to whole-body vibration. Suboccipital inhibition, whether applied alone or combined with myofascial relaxation, outperformed the placebo. Active release with fascial sliding was more effective than muscle energy techniques. The control groups showed no improvement, except with 30 s passive stretching.

**Table 3:** Characteristics of the studies.

Authors	Initial Sample (Women)	Intervention		Outcome Measures	Results
		Experimental Group	Control Group		
Aparicio et al. (2009) [37]	*n* = 70 (33%)	Suboccipital inhibition technique	Placebo	SLR; AKE; FFD; Algometry	EG obtained significant changes compared to CG for popliteal angle, SLR, FFD, and in the algometry of the right SM. No significant differences were obtained for the algometry of ST, BF, or left SM.
Azizi et al. (2021) [38]	*n* = 56 (100%)	Post-isometric relaxation technique (Lewit method)	Whole-body vibration	AKE; SR	EG and CG improved significantly in all tests. There was no significant difference between groups.
Chesterton et al. (2018) [35]	*n* = 20 (45%)	G1: central PA L5 mobilisation; G2: unilateral PA zygapophyseal L4/L5 mobilisation ipsilateral to the dominant limb	Placebo	AKE; ALF; EMG	EMG was lower and AKE and ALF values were higher after G1 and G2 treatments. EMG measures were lower, and AKE values were higher, after G2 treatment versus G1 treatment.
Chesterton et al. (2019) [40]	*n* = 24 (42%)	Unilateral PA zygapophyseal L4/L5 mobilisation ipsilateral to the dominant limb	No intervention	AKE; ALF	EG had a moderate effect on AKE and a moderate effect on ALF. AKE improvement became very small at 20 min after treatment and trivial after 60 min. For ALF, it became very small after 15 min and trivial after 25 min and 60 min.
Chesterton and Payton (2017) [34]	*n* = 38 (NP)	Central PA lumbar mobilisation to the L4 and L5 segments	Placebo	AKE; ALF; EMG	EG showed significant improvements in ALF and AKE.
					EMG activation of the ES and BF during lumbar flexion was reduced.
Joshi et al. (2018) [39]	*n* = 48 (67%)	Suboccipital inhibition + bilateral myofascial release in plantar fascia by therapist G1: by therapist; G2: self-applied	Bilateral passive hamstring stretch	AKE; SR	Hamstring flexibility improved in all three groups (pre- to post-intervention). CG showed additional benefits.
Khan et al. (2021) [41]	*n* = 60 (58%)	Post-isometric relaxation on the hamstring of dominant side	Active release technique on the hamstring of dominant side	SLR; AKE	EG and CG showed significant difference pre- to post-intervention. CG treatment was statistically significantly more effective than EG treatment.
Szlezak et al. (2011) [36]	*n* = 36 (47%)	G1: unilateral PA mobilisation from T12/L1 to L5/S1 zygapophyseal joints; G2: static stretch of the muscles of the posterior chain	Placebo	SLR	Only EG showed significant improvements in the SLR.

AKE: active knee extension test; ALF: active lumbar flexion test; BF: biceps femoris; CG: control group; EG: experimental group; EMG: electromyography; ES: erector spinae; FFD: fingers–floor distance test; G1: group 1; G2: group 2; PA: posterior–anterior; SLR: straight leg raise test; SM: semimembranosus; SR: sit-and-reach test; ST: semitendinosus.

## 4. Discussion

The aim of this systematic review was to analyse the scientific evidence on the efficacy of osteopathic techniques to improve hamstring flexibility in people with SHS. The papers incorporated into this review reported positive outcomes in enhancing hamstring flexibility through various osteopathic techniques [34,35,36,37,38,39,40,41].

The enhancements observed in muscle energy technique interventions [38,41] could be attributed to viscoelastic modifications (including mechanical factors and changes in the stretch tolerance threshold) and neurophysiological shifts (such as the inhibition reflex of the Golgi apparatus at the tendon level) that this particular technique induces in the muscle [44,45,46]. When the muscle contracts isometrically, as it does in this type of technique, the Golgi apparatus detects the increase in tension and triggers an autogenic inhibition response. This means that after a sustained muscle contraction, the Golgi tendon organs send signals to the central nervous system, causing a reflexive relaxation of the involved muscle. This process helps to reduce the activation of alpha motor neurons, which are responsible for muscle contraction, thereby allowing the muscle to relax and stretch more easily [44,45,46]. However, addressing muscle shortening through passive stretching techniques could trigger or worsen a local inflammatory response and might even lead to a more intense defensive muscle spasm, resulting in the opposite effect to what is intended [47]. This is another argument that strengthens the recommendation to use muscle energy techniques as a safe and effective method for improving flexibility, in contrast to other traditional stretching techniques, such as passive stretches [40,41,48,49,50].

Along the same lines, the improvements noted in articles employing articulatory techniques [34,35,36,39] can be justified as joint mobilisations are known to enhance neuromuscular spindle activity [51,52,53] and stimulate the Golgi tendon organ, leading to reflex inhibition of the muscle [54]. Moreover, lumbar mobilisations stimulate the periaqueductal gray matter and reduce the excitability of dorsal horn cells [55,56], along with displaying a transient inhibition of alpha motor neuron excitability via Hoffmann’s reflex. This results in a decrease in protective muscle defence, leading to an increase in joint range [51]. The Hoffmann reflex supports the feasibility of treating distal structures from a more proximal area [14,57], justifiable through the direct correlation between sympathetic arousal and pain modulation [58,59,60]. Other authors have obtained similar results to those found in this systematic review, analysing the effects on the hamstring muscles of therapeutic interventions performed on both the temporomandibular joint and the cervical region, also obtaining an increase in the flexibility of the muscles [30]. In addition, some studies have compared the beneficial effect of stretching techniques applied to the hamstring muscles on temporomandibular joint function [61,62].

Furthermore, the other muscle-aponeurotic techniques studied [37,38,40,41,43] base their effects on similar points, in turn justifying distal treatment. Suboccipital inhibition was found to be effective in elongating the hamstring muscles in three of the articles included in this review [37,40,43]. This is to be expected, as the suboccipital musculature is the region of the body with the highest concentration of neuromuscular spindles, and these are fundamental in the regulation of postural tone [37,63]. In particular, the posterior rectus minor muscle of the head (which has 36 neuromuscular spindles per gram) is known to be the main regulator of posture and the degree of cervical tension [37]. Because of this fact, suboccipital inhibition techniques can increase the elasticity of the hamstring muscles due to the relaxation of the superficial line of the back after inhibition of the suboccipital muscles and their relationship and connection with the dura mater, posture, and myofascial chain [37,63].

These findings are clinically relevant, as they imply functional improvements [12,50,64] and even injury prevention [65,66]. In functional terms, greater hamstring flexibility allows for an increased range of motion in the hip and knee joints, which facilitates everyday activities such as walking, running, jumping, and bending with greater efficiency and less restriction. Additionally, improved flexibility optimises the coordination and muscular balance between the hamstrings and antagonist muscles, such as the quadriceps, contributing to a more balanced and efficient posture, thereby enhancing overall physical performance [12,50,64].

Regarding injury prevention, flexible hamstrings reduce the risk of musculoskeletal injuries such as tendinopathies [19,20] or muscle tears by reducing stiffness and accumulated tension in the muscles [21]. Stiff and shortened muscles are more prone to injury during explosive or high-demand movements, especially in athletes [13]. By increasing flexibility, the muscle’s ability to absorb impacts and stretch under tension is enhanced, reducing the likelihood of muscle and tendon injuries [19,20,21]. Additionally, flexibility helps prevent muscular imbalances that can lead to postural compensations, overloading other structures, such as the back, which contributes to the prevention of related conditions like lower back pain [4,14,18,65,66].

One of the main limitations of this systematic review article is the high methodological heterogeneity observed in the included studies. There are significant variations in sample sizes, the osteopathic techniques used, and the methods for evaluating hamstring flexibility. This diversity makes it impossible to conduct a quantitative meta-analysis of the results, which could have provided a more robust and conclusive synthesis of the evidence. Therefore, it is recommended to interpret the findings with caution. Additionally, the lack of long-term follow-up in the studies limits the ability to assess the sustained effects of the interventions on hamstring flexibility.

Another relevant limitation is the suboptimal methodological quality of some of the included studies, as reflected in their scores on the PEDro scale, which average 4.8 points. The absence of blinding in most trials negatively impacts the rigour of the results and could introduce biases in the assessment of the effects of the interventions. Additionally, the presence of identified biases, particularly in outcome measurement and random sequence generation, may affect the validity of the findings of this systematic review. Studies such as that of Azizi et al. [38], with a high risk of bias in outcome measurement, could produce inaccurate data that either overestimate or underestimate the intervention effects. While some studies show moderate biases, such as Chesterton et al. [35] and Joshi et al. [39], their inclusion could impact the overall precision of results when combined with other studies. In contrast, studies with a low risk of bias, such as Aparicio et al. [37] and Szlezak et al. [36], strengthen the reliability of the overall conclusions. However, the cumulative impact of biases in some studies, particularly in outcome measurement and randomisation, suggests the need to interpret the findings cautiously and conduct sensitivity analyses to assess whether these limitations significantly affect the conclusions of the review [67]. On the other hand, it is worth mentioning that this is the first systematic review to analyse the effects of osteopathy in improving flexibility in patients with SHS. Finally, it should be noted that articles with the highest level of scientific evidence have been used, with research carried out up to March 2023.

Therefore, for future lines of research, it would be necessary to carry out new randomised controlled studies with higher methodological quality to support the analysis of these variables and with a longer follow-up. It would also be interesting to carry out research with stratification by sex and age, since both factors condition the flexibility of the hamstring musculature [1,4]. Further research is therefore needed to compare the effects of different interventions in order to develop appropriate treatment protocols for SHS patients.

## 5. Conclusions

The osteopathic techniques studied in relation to SHS (muscle energy techniques, joint mobilisations, and suboccipital inhibition) have shown a positive effect on improving the elasticity of the hamstring muscles. This effect can be explained by neurophysiological (inhibition of the Golgi apparatus, increased neuromuscular spindle activity, transient inhibition of alpha motor neuron excitability through the Hoffmann reflex) and structural factors (connection of the suboccipital muscles with the dura mater, the high concentration of neuromuscular spindles in the suboccipital musculature and its relationship with posture, and viscoelastic changes in the myofascial chain).

Nevertheless, further research with higher methodological quality and long-term follow-up is needed to determine whether these effects are sustained over time. Therefore, these findings should be interpreted with caution, and additional studies are required to support the efficacy of osteopathic treatment in short hamstring syndrome.

## Figures and Tables

**Table 1:** Methodological quality of the studies.

Author	1 *	2	3	4	5	6	7	8	9	10	11	Score
Aparicio et al. (2009) [37]	Yes	Yes	No	Yes	No	No	Yes	Yes	No	Yes	No	5
Azizi et al. (2021) [38]	Yes	Yes	Yes	Yes	No	No	No	Yes	No	Yes	No	5
Chesterton et al. (2018) [35]	Yes	Yes	No	Yes	No	No	Yes	Yes	No	Yes	No	5
Chesterton et al. (2019) [40]	Yes	Yes	Yes	Yes	No	No	Yes	Yes	No	Yes	No	6
Chesterton & Payton (2017) [34]	Yes	Yes	Yes	Yes	No	No	Yes	Yes	No	Yes	No	6
Joshi et al. (2018) [39]	Yes	Yes	No	Yes	No	No	Yes	Yes	Yes	Yes	Yes	7
Khan et al. (2021) [41]	Yes	Yes	No	Yes	No	No	No	Yes	No	Yes	No	4
Szlezak et al. (2011) [36]	Yes	Yes	No	Yes	Yes	No	No	Yes	No	Yes	No	5

Criteria: (1) Eligibility criteria specified; (2) subjects randomly allocated to groups; (3) concealed allocation; (4) groups were similar at baseline; (5) blinding of all subjects; (6) blinding of all therapists; (7) blinding of all assessors; (8) measures obtained from more than 85% of subjects allocated to groups; (9) subjects received treatment or control condition as allocated, or intention-to-treat analysis; (10) between-group statistical comparisons reported for at least one outcome; (11) both point measures and measures of variability were reported. * This item relates to external validity and therefore does not contribute to the total score.

**Table 2:** Risk of bias for included studies (RoB 2 tool results).

Authors	Random Sequence	Deviations from Intended Interventions	Missing Outcome Data	Measurement of the Outcome	Selection of the Reported Result	Overall
Aparicio et al. (2009) [37]	Low	Low	Low	Medium	Low	Medium
Azizi et al. (2021) [38]	Medium	Low	Low	High	Low	High
Chesterton et al. (2018) [35]	Medium	Low	Low	Medium	Low	Medium
Chesterton et al. (2019) [40]	Medium	Low	Low	Medium	Low	Medium
Chesterton and Payton (2017) [34]	Low	Low	Low	Low	Low	Low
Joshi et al. (2018) [39]	Medium	Low	Low	Medium	Low	Medium
Khan et al. (2021 [41]	Medium	Low	Medium	Medium	Low	Medium
Szlezak et al. (2011) [36]	Low	Low	Low	Low	Low	Low

## Data Availability

The data that support the findings of this study are available on request from the corresponding author, P.H.-L.

## Supplementary Materials

The following supporting information can be downloaded at: https://www.mdpi.com/article/10.3390/jcm13206076/s1. Table S1: Search strategy according to the focused question (PICOS). Table S2: PRISMA checklist.
