# Supplementary material for: Effectiveness of Osteopathic Treatment in Adults with Short Hamstring Syndrome: A Systematic Review
Source: J Clin Med. 2024 Oct 12;13(20):6076. doi: 10.3390/jcm13206076 (PMC11508461; doi:10.3390/jcm13206076)
Supplement: Supplementary file 1 [file jcm-13-06076-s001.zip › jcm-3221913-supplementary.pdf]

Journal of
Clinical Medicine

MDPI

Supplementary Table S1. Search strategy according to the focused question (PICOS).

Database	Search equation
**PubMed**	("Hamstring Muscles"[Mesh]) AND "Manipulation, Chiropractic"[Mesh]<br>("Hamstring Muscles"[Mesh]) AND "Manipulation, Orthopedic"[Mesh]<br>("Hamstring Muscles"[Mesh]) AND "Manipulation, Osteopathic"[Mesh]<br>("Hamstring Muscles"[Mesh]) AND "Manipulation, Spinal"[Mesh]<br>("Hamstring Muscles"[Mesh]) AND "Manual Therapy"<br>("Hamstring Muscles"[Mesh]) AND "Musculoskeletal Manipulations"[Mesh]<br>("Hamstring Muscles"[Mesh]) AND "Osteopathic Medicine"[Mesh]<br>("Hamstring Muscles"[Mesh] AND osteopath[Text Word])<br>("Hamstring Tendons"[Mesh]) AND "Manipulation, Chiropractic"[Mesh]<br>("Hamstring Tendons"[Mesh]) AND "Manipulation, Orthopedic"[Mesh]<br>("Hamstring Tendons"[Mesh]) AND "Manipulation, Osteopathic"[Mesh]<br>("Hamstring Tendons"[Mesh]) AND "Manipulation, Spinal"[Mesh]<br>("Hamstring Tendons"[Mesh]) AND "Manual therapy"<br>("Hamstring Tendons"[Mesh]) AND "Musculoskeletal Manipulations"[Mesh]<br>("Hamstring Tendons"[Mesh]) AND "Osteopathic Medicine"[Mesh]<br>("Hamstring Tendons"[Mesh]) AND osteopath[Text Word]
**Web of Science**	Hamstrings muscles (Topic) AND Manipulation, chiropractic (Topic)<br>Hamstrings muscles (Topic) AND Manipulation, Orthopedic (Topic)<br>Hamstrings muscles (Topic) AND Manipulation, Osteopathic (Topic)<br>Hamstrings muscles (Topic) AND Manipulation, Spinal (Topic)<br>Hamstrings muscles (Topic) AND Manual Therapy (Topic)<br>Hamstrings muscles (Topic) AND Musculoskeletal Manipulations (Topic)<br>Hamstrings muscles (Topic) AND Ostheopatic Medicine (Topic)<br>Hamstrings muscles (Topic) AND Ostheopath (Topic)<br>Hamstrings Tendons (Topic) and Manipulation, chiropractic (Topic)<br>Hamstrings Tendons (Topic) and Manipulation, orthopedic (Topic)<br>Hamstrings Tendons (Topic) and Manipulation, osteopathic (Topic)<br>Hamstrings Tendons (Topic) and Manipulation, spinal (Topic)<br>Hamstrings Tendons (Topic) AND Manual Therapy (Topic)<br>Hamstrings Tendons (Topic) AND Musculoskeletal Manipulations (Topic)<br>Hamstrings Tendons (Topic) and Ostheopatic Medicine (Topic)<br>Hamstrings Tendos (Topic) AND Ostheopath (Topic)
**Scopus**	TITLE-ABS-KEY ("Hamstring Muscles") AND TITLE-ABS-KEY ("Manipulation, Chiropractic")<br>TITLE-ABS-KEY ("Hamstring Muscles") AND TITLE-ABS-KEY ("Manipulation, Orthopedic")<br>TITLE-ABS-KEY ("Hamstring Muscles") AND TITLE-ABS-KEY ("Manipulation, Osteopathic"))<br>TITLE-ABS-KEY ("Hamstring Muscles") AND TITLE-ABS-KEY ("Manipulation, Spinal"))<br>TITLE-ABS-KEY ("Hamstring Muscles") AND TITLE-ABS-KEY ("Manual Therapy")<br>TITLE-ABS-KEY ("Hamstring Muscles") AND TITLE-ABS-KEY ("Musculoskeletal Manipulations")<br>TITLE-ABS-KEY ("Hamstring Muscles") AND TITLE-ABS-KEY ("Osteopathic Medicine")<br>TITLE-ABS-KEY ("Hamstring Muscles") AND TITLE-ABS-KEY (osteopath)<br>TITLE-ABS-KEY ("Hamstring Tendons") AND TITLE-ABS-KEY ("Manipulation, Chiropractic")<br>TITLE-ABS-KEY ("Hamstring Tendons") AND TITLE-ABS-KEY ("Manipulation, Orthopedic")<br>TITLE-ABS-KEY ("Hamstring Tendons") AND TITLE-ABS-KEY ("Manipulation, Osteopathic")<br>TITLE-ABS-KEY ("Hamstring Tendons") AND TITLE-ABS-KEY ("Manipulation, Spinal")<br>TITLE-ABS-KEY ("Hamstring Tendons") AND TITLE-ABS-KEY ("Manual Therapy")<br>TITLE-ABS-KEY ("Hamstring Tendons") AND TITLE-ABS-KEY ("Musculoskeletal Manipulations")<br>TITLE-ABS-KEY ("Hamstring Tendons") AND TITLE-ABS-KEY ("Osteopathic Medicine")<br>(TITLE-ABS-KEY ("Hamstring Tendons") AND TITLE-ABS-KEY (osteopath)
**Medline**	(MH "Hamstring Muscles") AND (MH “Manipulation, Chiropractic")<br>(MH "Hamstring Muscles") AND (MH "Manipulation, Orthopedic")<br>(MH "Hamstring Muscles") AND (MH "Manipulation, Osteopathic")<br>(MH "Hamstring Muscles") AND (MH "Manipulation, Spinal")<br>(MH "Hamstring Muscles") AND (MH "Musculoskeletal Manipulations")<br>(MH "Hamstring Muscles") AND (MH "Osteopathic Medicine")<br>(MH "Hamstring Muscles") AND (TX "Manual Therapy")<br>(MH "Hamstring Muscles") AND (TX "osteopath")<br>(MH "Hamstring Tendons") AND (MH “Manipulation, Chiropractic")<br>(MH "Hamstring Tendons") AND (MH "Manipulation, Orthopedic")<br>(MH "Hamstring Tendons") AND (MH "Manipulation, Osteopathic")<br>(MH "Hamstring Tendons") AND (MH "Manipulation, Spinal")<br>(MH "Hamstring Tendons") AND (MH "Musculoskeletal Manipulations")<br>(MH "Hamstring Tendons") AND (MH "Osteopathic Medicine")<br>(MH "Hamstring Tendons") AND (TX "Manual Therapy")<br>(MH "Hamstring Tendons") AND (TX "osteopath")
**Cinhal**	"hamstring tendons" AND "manipulation, spinal"<br>"hamstring tendons" AND "Musculoskeletal Manipulations"<br>"hamstring tendons" AND (MH "Manipulation, Chiropractic")<br>"hamstring tendons" AND (MH "Manipulation, Orthopedic")<br>"hamstring tendons" AND (MH "Manipulation, Osteopathic")<br>"hamstring tendons" AND (MH "Manual Therapy")

	"hamstring tendons" AND (MH "Osteopathic Medicine")<br>"hamstring tendons" AND "osteopath"<br>(MH "Hamstring Muscles") AND "Manipulation, Spinal"<br>(MH "Hamstring Muscles") AND "Musculoskeletal Manipulations"<br>(MH "Hamstring Muscles") AND (MH "Manipulation, Chiropractic")<br>(MH "Hamstring Muscles") AND (MH "Manipulation, Orthopedic")<br>(MH "Hamstring Muscles") AND (MH "Manipulation, Osteopathic")<br>(MH "Hamstring Muscles") AND (MH "Manual Therapy")<br>(MH "Hamstring Muscles") AND (MH "Osteopathic Medicine")<br>(MH "Hamstring Muscles") AND "osteopath"
**SPORTDiscuss**	DE "HAMSTRING muscle" AND Manipulation Chiropractic<br>DE "HAMSTRING muscle" AND Manipulation Orthopedic<br>DE "HAMSTRING muscle" AND Manipulation Osteopathic<br>DE "HAMSTRING muscle" AND Manipulation Spinal<br>DE "HAMSTRING muscle" AND Manual Therapy<br>DE "HAMSTRING muscle" AND Muskuloeskeletal Manipulation<br>DE "HAMSTRING muscle" AND osteopathic Medicine<br>DE "HAMSTRING muscle" AND osteopathç<br>HAMSTRING Tendons AND manipulation, Chiropractic<br>HAMSTRING Tendons AND manipulation, Orthopedic<br>HAMSTRING Tendons AND manipulation, osteopathic<br>HAMSTRING Tendons AND manipulation, Spinal<br>HAMSTRING Tendons AND Manual Therapy<br>HAMSTRING Tendons AND Musculoeskeletal Manipulation<br>HAMSTRING Tendons AND osteopathic Medicine<br>HAMSTRING Tendons AND osteopath

Supplementary Table S2. PRISMA checklist.

Section and Topic	Item #	Checklist item	Location where item is reported
**TITLE**			
Title	1	Identify the report as a systematic review.	1 pag
**ABSTRACT**			
Abstract	2	See the PRISMA 2020 for Abstracts checklist.	1 pag
**INTRODUCTION**			
Rationale	3	Describe the rationale for the review in the context of existing knowledge.	2-3 pag
Objectives	4	Provide an explicit statement of the objective(s) or question(s) the review addresses.	2-3 pag
**METHODS**			
Eligibility criteria	5	Specify the inclusion and exclusion criteria for the review and how studies were grouped for the syntheses.	3-4 pag
Information sources	6	Specify all databases, registers, websites, organisations, reference lists and other sources searched or consulted to identify studies. Specify the date when each source was last searched or consulted.	3 pag
Search strategy	7	Present the full search strategies for all databases, registers and websites, including any filters and limits used.	2 pag-Table suplmentary1
Selection process	8	Specify the methods used to decide whether a study met the inclusion criteria of the review, including how many reviewers screened each record and each report retrieved, whether they worked independently, and if applicable, details of automation tools used in the process.	4 pag
Data collection process	9	Specify the methods used to collect data from reports, including how many reviewers collected data from each report, whether they worked independently, any processes for obtaining or confirming data from study investigators, and if applicable, details of automation tools used in the process.	4-5 pag
Data items	10a	List and define all outcomes for which data were sought. Specify whether all results that were compatible with each outcome domain in each study were sought (e.g. for all measures, time points, analyses), and if not, the methods used to decide which results to collect.	4 pag
	10b	List and define all other variables for which data were sought (e.g. participant and intervention characteristics, funding sources). Describe any assumptions made about any missing or unclear information.	4 pag
Study risk of bias assessment	11	Specify the methods used to assess risk of bias in the included studies, including details of the tool(s) used, how many reviewers assessed each study and whether they worked independently, and if applicable, details of automation tools used in the process.	4 pag
Effect measures	12	Specify for each outcome the effect measure(s) (e.g. risk ratio, mean difference) used in the synthesis or presentation of results.	Not applicable
Synthesis methods	13a	Describe the processes used to decide which studies were eligible for each synthesis (e.g. tabulating the study intervention characteristics and comparing against the planned groups for each synthesis (item #5)).	4-5 pag
	13b	Describe any methods required to prepare the data for presentation or synthesis, such as handling of missing summary statistics, or data conversions.	4-5 pag
	13c	Describe any methods used to tabulate or visually display results of individual studies and syntheses.	4-5 pag
	13d	Describe any methods used to synthesize results and provide a rationale for the choice(s). If meta-analysis was performed, describe the model(s), method(s) to identify the presence and extent of statistical heterogeneity, and software package(s) used.	4-5 pag
	13e	Describe any methods used to explore possible causes of heterogeneity among study results (e.g. subgroup analysis, meta-regression).	4-5 pag
	13f	Describe any sensitivity analyses conducted to assess robustness of the synthesized results.	Not applicable

Reporting bias assessment	14	Describe any methods used to assess risk of bias due to missing results in a synthesis (arising from reporting biases).	4 pag
Certainty assessment	15	Describe any methods used to assess certainty (or confidence) in the body of evidence for an outcome.	Not applicable
**RESULTS**			
Study selection	16a	Describe the results of the search and selection process, from the number of records identified in the search to the number of studies included in the review, ideally using a flow diagram.	Figure1- 3 pag
	16b	Cite studies that might appear to meet the inclusion criteria, but which were excluded, and explain why they were excluded.	Figure 1 - 3 pag
Study characteristics	17	Cite each included study and present its characteristics.	Table 3
Risk of bias in studies	18	Present assessments of risk of bias for each included study.	Figure 2 – 5 pag
Results of individual studies	19	For all outcomes, present, for each study: (a) summary statistics for each group (where appropriate) and (b) an effect estimate and its precision (e.g. confidence/credible interval), ideally using structured tables or plots.	Table 3
Results of syntheses	20a	For each synthesis, briefly summarise the characteristics and risk of bias among contributing studies.	Figure 2 – 3 pag
	20b	Present results of all statistical syntheses conducted. If meta-analysis was done, present for each the summary estimate and its precision (e.g. confidence/credible interval) and measures of statistical heterogeneity. If comparing groups, describe the direction of the effect.	Not applicable
	20c	Present results of all investigations of possible causes of heterogeneity among study results.	Not applicable
	20d	Present results of all sensitivity analyses conducted to assess the robustness of the synthesized results.	Not applicable
Reporting biases	21	Present assessments of risk of bias due to missing results (arising from reporting biases) for each synthesis assessed.	Table 2
Certainty of evidence	22	Present assessments of certainty (or confidence) in the body of evidence for each outcome assessed.	Not applicable
**DISCUSSION**			
Discussion	23a	Provide a general interpretation of the results in the context of other evidence.	11-13 pag
	23b	Discuss any limitations of the evidence included in the review.	11-13 pag
	23c	Discuss any limitations of the review processes used.	13 pag
	23d	Discuss implications of the results for practice, policy, and future research.	13 pag
**OTHER INFORMATION**			
Registration and protocol	24a	Provide registration information for the review, including register name and registration number, or state that the review was not registered.	3 pag
	24b	Indicate where the review protocol can be accessed, or state that a protocol was not prepared.	3 pag
	24c	Describe and explain any amendments to information provided at registration or in the protocol.	3 pag
Support	25	Describe sources of financial or non-financial support for the review, and the role of the funders or sponsors in the review.	14 pag
Competing interests	26	Declare any competing interests of review authors.	14 pag
Availability of data, code and other materials	27	Report which of the following are publicly available and where they can be found: template data collection forms; data extracted from included studies; data used for all analyses; analytic code; any other materials used in the review.	14 pag
